# Supplementary material for: Do people imitate when making decisions? Evidence from a spatial Prisoner’s Dilemma experiment
Source: R Soc Open Sci. 2020 Jul 29;7(7):200618. doi: 10.1098/rsos.200618 (PMC7428279; doi:10.1098/rsos.200618)
Supplement: Supplementary Material for Do people imitate when making a decision [file rsos200618supp1.pdf]

Supplementary Material for : Do people imitate when making decisions? - evidence from a spatial prisoner's dilemma experiment

Jelena Grujić [1,2] and Tom Lenaerts [1,2]

[1]AI Lab, Vrije Universiteit Brussel, Brussels, Belgium
[2]MLG, Université Libre de Bruxelles, Brussels, Belgium

June 22, 2020

1 Instructions and tests for experiments

The full instructions for each treatment are available in two separate files:

- Instructions_without_info_en.pdf
- Instructions_with_info_en.pdf

Both files are available within the Dryad dataset https://datadryad.org/stash/share/VDXat91y1yw–CdHofjKBMwjLpZaYEjJhVIyCDb4LeY. The participants all read these instructions on the screens, while having also this information available on paper for later consultation throughout the experiment.

Once they completed reading the instructions, the participants were asked to answer what would be their payoff in the situations presented in Figures 1 and 2. The examples are chosen to be as neutral as possible so they would not frame the behaviour of the participants. We statistically tested, as explained in the main text, whether players who saw one set of examples did not act differently from the players who saw the other set.

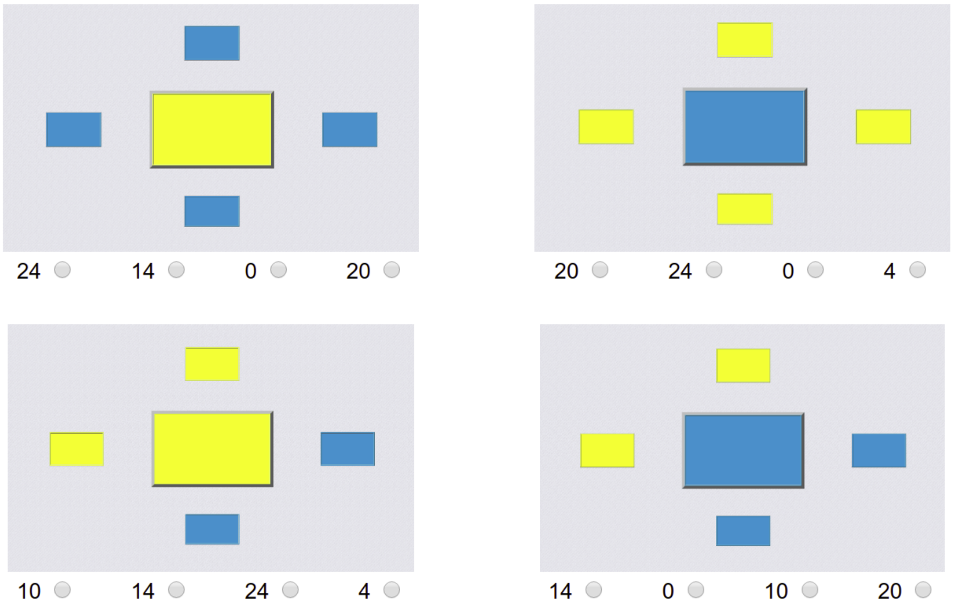

Figure 1: The test examples in the first three sessions in both TWO and TWI treatments - Examples 0 in the Data Set.

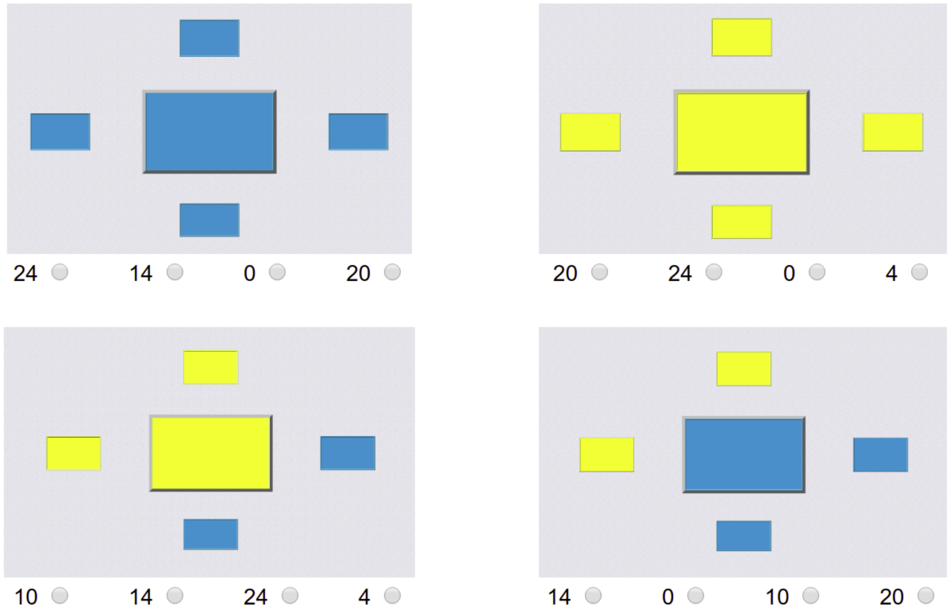

Figure 2: The test examples in the last session two session in TWI and the last session in TWO treatment - Examples 1 in the Data Set.

Additionally, the participants were asked to answer the following two yes/no questions to make sure they understood the game:

- Are you playing with the same neighbours in every round?
- Are the rules of the game the same for all the players?

2 More gender statistics

We assured also that both treatments were gender-balanced. The detailed information per treatment is shown in the table below.

	**TWI**	**TWO**	**Total**
female	0.4	0.53	0.46
male	0.6	0.47	0.54
average age (SD)	24 (4.5)	23.7 (4.4)	23.9 (4.4)

3 Reaction Times

The experiment reported in this paper takes more rounds than most PD experiments: People participated for 50 rounds as opposed to only 10 or 15 (They were informed that the numbers of rounds was undetermined but that the entire experiment would take less than an hour). As in such longer experiments people may get distracted and wait too long to make their choice, the decision was made to introduce a timer on the screen, counting down from 30 to 0 seconds. The timer ensures that the pace is kept so that the overall experiment can finish within the hour. If a person could cause long delays in the game, then other participants may get bored and irritated, and change their behaviour accordingly. Note again that even when the timer reached 0, nothing would happen (e.g. no automatic selection of an action), except that the participant would be urged to make choice. As was shown in [1], the reaction time distribution decreases

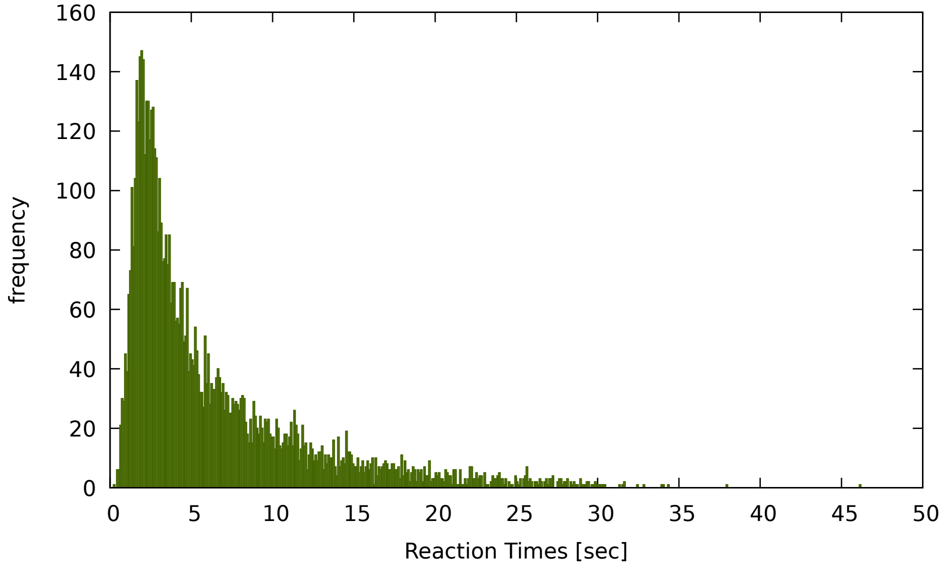

Figure 3: The distribution of Reaction Times in the Data Set. We see that the typical Reaction time is an order of magnitude shorter than the suggested time limit of 30s.

exponentially and participants take an order of magnitude less time than the limit of 30 seconds. This can also be observed in the reaction time distribution of the current experiment as can be seen in Figure 3.

References and Notes

[1] Gallotti R, Grujić J. A quantitative description of the transition between intuitive altruism and rational deliberation in iterated Prisoner?s Dilemma experiments. Scientific reports. 2019;9:17046.
